# Modified Palomo Procedure Is an Effective Intervention for Improving Serum Testosterone Levels and Semen Parameters in Patients With Varicocele: A Prospective Study

**DOI:** 10.7759/cureus.35252

**Published:** 2023-02-21

**Authors:** Dheer S Kalwaniya, Aditya Tolat, Devender Kumar, Vakulabharanam Naga Rohith

**Affiliations:** 1 General Surgery, Vardhman Mahavir Medical College and Safdarjung Hospital, New Delhi, IND; 2 General Surgery, Safdarjung Hospital, New Delhi, IND

**Keywords:** modified palomos' procedure, testis, semen parameters, serum testosterone, varicocele treatment

## Abstract

Background: A varicocele can be defined as an abnormal venous dilatation and/or tortuosity of the pampiniform plexus. It is generally reported that varicoceles are present in 15% of the general male population and 35% of men as a cause of primary infertility and in up to 80% of men as a cause of secondary infertility. Differences in venous drainage anatomy between the left and right internal spermatic veins, venous valve incompetence resulting in venous blood reflux, and increased hydrostatic pressure are the most commonly cited theories. Various surgical and non-surgical techniques are in use for treating patients with varicocele. Here we used a modified Palamo procedure to treat the patients and observed the outcome.

Methodology: A total of 40 patients with varicocele were recruited for the study. A preoperative evaluation, along with serum testosterone levels and semen analysis, was done. A modified Palomo technique was used to treat varicocele. A postoperative follow-up with serum testosterone levels and semen analysis was done to observe improvement.

Results: The mean (±SD) left testis size, right testis size, testosterone, sperm concentration, sperm vitality and sperm progressive motility were found statistically significantly higher in patients after surgery as compared to patients before surgery (p<0.05). However, there was statistically insignificant mean difference in semen volume between patients before surgery and patients after surgery (p>0.05).

Conclusion: Modified Palomo procedure can be used to treat varicocele with good improvement in serum testosterone levels and semen parameters.

## Introduction

A varicocele can be defined as an abnormal venous dilatation and/or tortuosity of the pampiniform plexus in the scrotum. Although varicoceles are almost always larger and more common on the left side, up to 50% of men with varicoceles have bilateral varicoceles [1]. It is generally reported that varicoceles are present in 15% of the general male population, in 35% of men with primary infertility, and up to 80% of men with secondary infertility [2]. Varicocele is thought to have a multi-factorial etiology. Differences in venous drainage anatomy between the left and right internal spermatic veins, venous valve incompetence resulting in venous blood reflux, and increased hydrostatic pressure are the most commonly cited theories. Physical exertion during puberty may lead to the development of varicocele, whereas physical exertion at a later age can aggravate the condition but does not modify the prevalence of varicocele [3].

Previous studies have found a link between varicocele and decreased male fertility potential (e.g., poor semen parameters and infertility). Several mechanisms have been proposed by researchers to explain the pathophysiology of varicocele. Scrotal hyperthermia likely represents the primary mechanism by which a varicocele affects endocrine function and spermatogenesis [4,5].

Varicoceles are the commonly seen and correctable cause of male factor infertility [6]. Based on current evidence, it is the practice guideline of both the American Urological Association and the American Society for Reproductive Medicine (ASRM) that correction of a varicocele should be offered to infertile men with palpable lesions and one or more abnormal semen parameters [7].

While the majority of varicoceles are asymptomatic, pain can occur in up to 10% of cases. Varicocele-related pain is typically mild, dull aching, and is usually limited to the testicle or spermatic cord. While chronic, severe pain is an additional indication of repair, because of the high incidental prevalence of varicoceles in the general population, a careful evaluation to rule out other etiologies, as well as a period of conservative management, are required before surgical treatment [8]. 

If a candidate is diagnosed with varicocele, he will be declared medically unfit for recruitment to the armed forces. However, if a varicocele is corrected without any complications, the candidate can be declared fit. Thus, recruitment to the armed forces can also be an indication of varicocele repair.

For the treatment of varicocele, a variety of surgical and nonsurgical methods have been recommended, including laparoscopic and microsurgical (inguinal or subinguinal) varicocelectomy, retroperitoneal Palomo surgery as well as percutaneous radiologic techniques (embolization or sclerotherapy).

Mellinger BC et al. studied that laparoscopic varix ligation, modified Palomo or high ligation, transinguinal or subinguinal with or without magnification, and transvenous percutaneous occlusion are a few techniques used to do varicocelectomy. All techniques appear to produce equivalent improvements in semen quality and rates of pregnancy in the patient's female spouse [9].

Hing Du et al. studied various surgical techniques to treat varicocele and their outcome with respect to male infertility and concluded that the microsurgical technique is superior to treating infertility in patients with varicocele [10].

There is a paucity of studies that observed the modified Palomo technique as an effective approach to treating male infertility in patients with varicocele. In this study, we used a modified Palomo procedure to treat patients with varicocele and noted the outcome of improvement in serum testosterone levels and semen parameters.

## Materials and methods

The study was conducted in the department of general surgery at VMMC & Safdarjung Hospital, New Delhi, from June 2021 to June 2022 after obtaining approval from the Institutional Ethics Committee (approval number of IEC/VMMC/SJH/2022-14/CC-223). In this study, we aimed to investigate the effectiveness of the modified Palomo procedure in improving serum testosterone levels and semen parameters in patients with varicocele.

Inclusion and exclusion criteria: 40 patients having either unilateral or bilateral varicocele were recruited for the study after their written informed consent. These patients had one of the three indications for varicocele repair: symptoms (pain), infertility, or recruitment in the armed forces.

Patients with co-morbidities and with ages >40 years were excluded from the study.

Methodology: History was taken, the examination done, and a complete pre-operative evaluation was done, including semen analysis, serum testosterone levels, and USG arterial & venous Doppler of the scrotum. The testicular size was determined using ultrasonography. Normal testis size was considered as 14-18cc [11]. Normal serum testosterone levels were considered as > 300 ng/dL. Normal semen parameters were considered according to the WHO criteria 2010 (Appendix 1). Varicocele was graded according to Dubins’ and Amelars’ criteria: grade I: small, only detected during Valsalva maneuver (upright position); grade II: moderate, easily palpated without Valsalva maneuver; and grade III: large, causing visible bulging of the scrotal skin (``sac full of worms''). All patients were operated on using the modified Palomo technique through a left (or right) lower abdominal quadrant muscle-splitting incision, the internal spermatic vessels were dissected, and the internal spermatic veins ligated close to the internal inguinal ring with sparing of the testicular artery and lymphatics [12]. Postoperatively, patients were followed up at three months with serum testosterone levels and semen analysis and at six months to look for any postoperative hydrocele.

Statistical analysis was carried out by frequency and proportion for categorical variables. Continuous variables were presented as mean (±standard deviation). The Chi-square test was used to test the statistical significance of cross-tabulation between categorical variables. An independent t-test was used to compare the mean (±standard deviation) of continuous variables between the two groups. Paired t-test was used to compare the mean (±standard deviation) between pairs of measurement. The Pearson correlation test was used to assess the correlation between two continuous variables.

P value < 0.05 was considered statistically significant. RStudio Desktop Version 2022.07.0+492 was used for statistical analysis. (Reference: RStudio Team (2022). RStudio: Integrated Development for R. RStudio, PBC, Boston, MA URL http://www.rstudio.com/).

## Results

A total of 40 patients were included in the study. The mean (±SD) age of the study population was 25.73 (±4.84) years, among which 52.5% (n=21) were between 19 and 24 years, 32.5% (n=13) were between 25 and 35 years, and 15% (n=6) were between 31 and 40 years. 37.5% (n=15) had bilateral varicoceles and 62.5% (n=25) had unilateral varicocele. Among 25 patients with unilateral varicocele, 23 had left side affected, and two had right side affected. Among 15 bilateral varicoceles, 10 were graded I, 6 were graded II, and 14 were graded III. Among 25 unilateral varicocele, nine were graded II, and 16 were graded III. The indication for surgery was infertility for symptomatic for 60% (n=24) of the patients, recruitment in government jobs for 22.5% (n=9) of the patients, and infertility for 17.5% (n=7) of the patients (Table 1).

**Table 1:** Background characteristics of the study population

Background characteristics		Frequency	Percentage
Age group	19 to 24 years	21	52.5%
	25 to 30 years	13	32.5%
	31 to 40 years	6	15.0%
Laterality	Bilateral	15	37.5%
	Unilateral	25	62.5%
Indication for surgery	Symptomatic	24	60.0%
	Recruitment in government job	9	22.5%
	Infertility	7	17.5%

In unilateral cases, there was a statistically insignificant mean difference in left testis size, right testis size, testosterone, semen volume, sperm concentration, sperm vitality, and sperm progressive motility according to varicocele grade (p>0.05). The correlation of testosterone with semen volume, sperm concentration, sperm vitality, and sperm progressive motility decreased after surgery. There was an insignificant correlation between testosterone and semen volume after surgery (p>0.05). There was a moderate and positive correlation between testosterone with sperm concentration and sperm vitality after surgery (p<0.05). There was a high and positive correlation of testosterone with sperm progressive motility after surgery (p<0.05) (Table 2).

**Table 2:** Comparison of testes size, testosterone and semen analysis parameters according to grade in unilateral cases. Mean difference = II minus III

Variables	Varicocele grade		Mean difference (95% CI)	P-value
	II (Mean ± SD)	III (Mean ± SD)		
Left Testis size (cc)	15.64±0.74	14.69±1.28	0.95(-0.02,1.92)	0.054
Right Testis size (cc)	16.37±0.44	15.75±0.97	0.62(-0.1,1.33)	0.087
Testosterone (ng/dL)	657.56±210.33	528.88±129.34	128.68(-40.38,297.74)	0.123
Semen volume (ml)	3.42±0.61	2.99±0.72	0.43(-0.16,1.03)	0.143
Sperm concentration (million/ml)	38.00±9.35	33.56±12.14	4.44(-5.26,14.14)	0.354
Sperm vitality (%)	67.67±6.26	68.13±5.49	-0.46(-5.43,4.52)	0.850
Sperm progressive motility (%)	48.33±6.87	45.56±6.36	2.77(-2.87,8.41)	0.320

The mean (±SD) left testis size, right testis size, testosterone, sperm concentration, sperm vitality, and sperm progressive motility were found statistically significantly higher in patients with unilateral varicocele as compared to patients with bilateral varicocele (p<0.05). However, there was a statistically insignificant mean difference in semen volume between patients with unilateral varicocele and bilateral varicocele (p>0.05). On average, left testis size was higher in patients with unilateral varicocele by 1.74 cc (95% CI: 0.64, 2.83) as compared to patients with bilateral varicocele. Right testis size was higher in patients with unilateral varicocele by 1.45 cc (95% CI: 0.55, 2.35) on average as compared to patients with bilateral varicocele. On average, testosterone was higher in patients with unilateral varicocele by 182.13 ng/dL (95% CI: 57.59, 306.68) as compared to patients with bilateral varicocele. Sperm concentration was higher in patients with unilateral varicocele by 13.23 million/ml (95% CI: 5.58, 20.87) on average as compared to patients with bilateral varicocele. Sperm vitality was higher in patients with unilateral varicocele by 12.29% (95% CI: 5.26, 19.32) on average as compared to patients with bilateral varicocele. Sperm progressive motility was higher in patients with unilateral varicocele by 10.49% (95% CI: 4.60, 16.39) on average as compared to patients with bilateral varicocele (Table 3).

**Table 3:** Comparison of testes size, testosterone, and semen analysis parameters according to laterality.

Variables	Laterality		Mean difference (95% CI)	P-value
	Unilateral (Mean ± SD)	Bilateral (Mean ± SD)		
Left Testis size (cc)	15.04±1.19	13.30±1.82	1.74(0.64,2.83)	0.003
Right Testis size (cc)	15.97±0.86	14.53±1.53	1.45(0.55,2.35)	0.003
Testosterone (ng/dL)	575.20±170.81	393.07±215.18	182.13(57.59,306.68)	0.005
Semen volume (ml)	3.14±0.71	2.97±0.69	0.17(-0.29,0.63)	0.461
Sperm concentration (million/ml)	35.16±11.23	21.93±12.13	13.23(5.58,20.87)	0.001
Sperm vitality (%)	67.96±5.65	55.67±12.18	12.29(5.26,19.32)	0.002
Sperm progressive motility (%)	46.56±6.55	36.07±9.76	10.49(4.60,16.39)	0.001

There was a low and positive correlation between testosterone and semen volume before surgery (p<0.05). There was a high and positive correlation of testosterone with sperm concentration, sperm vitality, and sperm progressive motility before surgery (p<0.05) (Table 4).

**Table 4:** Correlation between testosterone and semen analysis parameters (before surgery).

Variables (Before)	Correlation coefficient (95% CI)	P-value
Testosterone and Semen volume	0.330(0.020,0.582)	0.038
Testosterone and Sperm concentration	0.636(0.404,0.791)	<0.001
Testosterone and Sperm vitality	0.683(0.472,0.820)	<0.001
Testosterone and Sperm progressive motility	0.734(0.547,0.851)	<0.001

The correlation of testosterone with semen volume, sperm concentration, sperm vitality, and sperm progressive motility decreased after surgery. There was an insignificant correlation between testosterone and semen volume after surgery (p>0.05). There was a moderate and positive correlation between testosterone with sperm concentration and sperm vitality after surgery (p<0.05). There was a high and positive correlation of testosterone with sperm progressive motility after surgery (p<0.05) (Table 5).

**Table 5:** Correlation between testosterone and semen analysis parameters (After surgery).

Variables (After)	Correlation coefficient (95% CI)	P-value
Testosterone and Semen volume	0.066(-0.250,0.370)	0.684
Testosterone and Sperm concentration	0.400(0.101,0.633)	0.010
Testosterone and Sperm vitality	0.460(0.173,0.675)	0.003
Testosterone and Sperm progressive motility	0.603(0.358,0.770)	<0.001

The mean (±SD) left testis size, right testis size, testosterone, sperm concentration, sperm vitality, and sperm progressive motility were found statistically significantly higher in patients after surgery as compared to patients before surgery (p<0.05). However, there was a statistically insignificant mean difference in semen volume between patients before surgery and patients after surgery (p>0.05). On average, left testis size was higher in patients after surgery by 0.20 cc (95% CI: 0.13, 0.27) as compared to patients before surgery. Right testis size was higher in patients after surgery by 0.05 cc (95% CI: 0.02, 0.100) on average as compared to patients before surgery. On average, testosterone was higher in patients after surgery by 78.25 ng/dL (95% CI: 32.15, 124.35) as compared to patients before surgery. Sperm concentration was higher in patients after surgery by 5.65 million/ml (95% CI: 4.66, 6.64) on average as compared to patients before surgery. Sperm vitality was higher in patients after surgery by 6.35% (95% CI: 4.77, 7.93) on average as compared to patients before surgery. Sperm progressive motility was higher in patients after surgery by 5.30% (95% CI: 4.37, 6.23) on average as compared to patients before surgery (Table 6).

**Table 6:** Comparison of testes size, testosterone, and semen analysis parameters before and after surgery.

Variables	Before surgery (Mean ± SD)	After surgery (Mean ± SD)	Mean difference (95% CI)	P-value
Left Testis size (cc)	14.39±1.67	14.59±1.60	0.20(0.13,0.27)	<0.001
Right Testis size (cc)	15.43±1.34	15.48±1.29	0.05(0.002,0.100)	0.043
Testosterone (ng/dL)	506.9±206.28	585.15±203.55	78.25(32.15,124.35)	0.001
Semen volume (ml)	3.08±0.70	3.05±0.71	-0.03(-0.12,0.07)	0.570
Sperm concentration (million/ml)	30.20±13.13	35.85±11.87	5.65(4.66,6.64)	<0.001
Sperm vitality (%)	63.35±10.45	69.70±6.26	6.35(4.77,7.93)	<0.001
Sperm progressive motility (%)	42.63±9.33	47.93±8.23	5.30(4.37,6.23)	<0.001

## Discussion

Varicocele is a disorder that develops usually after adolescence and may progress thereafter. Evidently, the mean age of patients in our study was 25.73 years. Most of these patients might have had an onset of varicocele at adolescence which progressed with age till it caused symptoms of infertility or trouble in recruitment [13]. 

It is established that varicocele is more common on the left side due to the attachment of the left gonadal vein to the left renal vein at right angles. Similarly, in our study, 25 patients had unilateral varicocele, 23 had left side affected, and two had right side affected, 15 patients had a bilateral varicocele. Isolated right-sided varicocele was aggressively investigated to rule out any intra-abdominal mass lesion.

Varicocele can lead to testicular atrophy. Although varicocele repair does not significantly increase the testis size in adults, it may lead to catch-up growth if repaired in adolescence [14]. In our study, there was a slight increase in testicular volume postoperatively.

There have been conflicting results in the literature regarding the change in serum testosterone levels after varicocele repair. Res orlu et al. published a study to suggest no change in serum testosterone after varicocele repair [15]. Hsiao et al. reported that varicocelectomy is associated with a significant increase in testosterone levels across all age groups [16]. In our study, there was a significant increase in serum testosterone levels in patients where it was below the lower limit as well as in patients where it was in the normal range.

In our study, varicocele repair led to an increase in sperm concentration, sperm vitality, and sperm progressive motility. Similar results have been reported by a meta-analysis done by Baazeem A et al. [17]. There was no change in semen volume before and after varicocele repair. Whether improvement in semen parameters led to conception would require a longer follow-up and was not possible due to the time-bound nature of the study.

Many of the previous studies showed conflicting results regarding the correlation between serum testosterone levels and sperm count and sperm morphology. Lenau H et al. demonstrated a direct correlation between serum testosterone levels and sperm counts [18]. Netter A et al. demonstrated no correlation between serum testosterone level and sperm count [19].

In our study, there was a positive correlation between sperm concentration, sperm vitality, and sperm progressive motility before and after surgery.

## Conclusions

The modified Palomo procedure is a reliable technique for treating varicocele and also for improvement in serum testosterone levels and seminal parameters in patients with varicocele.

## Appendices

The criteria of normal semen parameters as defined by WHO in 2010 is shown in Table 7.

**Table 7:** WHO criteria of normal semen parameters 2010

Parameters	Lower reference limit
Semen volume (ml)	1.5
Sperm concentration (10^6^/ml)	15
Total sperm number (10^6^/ejaculation)	39
Progressive motility (%)	32
Total Motility (%)	40
Vitality (%)	58
Sperm morphology (NF,%)	4
pH	>/=7.2
Leucocytes (10^6^/ml)	<1
Immunobead test (%)	<50
